# Rheology and Stability of Tunicate Cellulose Nanocrystal-Based Pickering Emulsions: Role of pH, Concentration, and Emulsification Method

**DOI:** 10.3390/foods15030509

**Published:** 2026-02-01

**Authors:** Sumana Majumder, Matthew J. Dunlop, Bishnu Acharya, Supratim Ghosh

**Affiliations:** 1 Department of Chemical and Biological Engineering, College of Engineering, University of Saskatchewan, Saskatoon, SK S7N 5E5, Canada; 2 Department of Chemistry, University of Prince Edward Island, Charlottetown, PE C1A 4P3, Canada; 3 Department of Food and Bioproduct Sciences, College of Agriculture & Bioresources, University of Saskatchewan, Saskatoon, SK S7N 5A8, Canada

**Keywords:** high aspect-ratio CNC, marine biomass-derived nanomaterials, emulsion stability, pH-dependent droplet size, zeta potential, high-shear homogenization, ultrasonication, detachment energy, rheology, gel strength, pH-dependent wettability

## Abstract

Tunicate (marine invertebrates)-derived cellulose nanocrystals (T-CNC) possess unique structural and physicochemical properties compared to other wood-based CNCs. This study aimed to characterize and utilize T-CNC as a stabilizer in Pickering emulsion (PE), highlighting a sustainable alternative to conventional surfactant-based emulsifiers. Characterization of T-CNC revealed a rod-shaped morphology with dimensions of 1694 ± 925 nm in length and 13 ± 3 nm in width, resulting in an aspect ratio of 122 ± 45, and high crystallinity (87.6%). Its zeta potential ranged from −4.4 to −45.5 mV across pH 2–10 and contact angles <50° indicate strong water wettability. T-CNC at 0.2%, 0.3%, and 0.4% (*w*/*w*) at pH 3 and 5 was used to prepare 20 wt% oil-in-water PE using a high-shear homogenizer followed by ultrasonication. Ultrasonication significantly improved the emulsion stability compared to only high-shear homogenization, decreasing droplet size by 31.4–50.8% and 55.7–89.3% for pH 3 and pH 5, respectively. PEs developed at pH 3 demonstrated smaller droplet sizes, better stability with minimal coalescence after 7 days, and enhanced gel-like rheological behaviour compared to PEs at pH 5, which displayed flocculation and coalescence. The gel strength of the pH 3 PEs increased with T-CNC concentration, as evidenced by progressively denser droplet packing, consistent with stronger interfacial anchoring (higher detachment energy) and reduced coalescence. This study underscores T-CNC’s superior efficiency in stabilizing PEs at low concentrations, offering a green, high-performance solution for food, cosmetic, and pharmaceutical applications.

## 1. Introduction

An emulsion is a mixture of two immiscible liquids (oil and water), generally stabilized by surfactants. Synthetic surfactant-based emulsions are often associated with various short-term and long-term health and environmental risks [1]. In this context, biobased approaches to emulsion stabilization can be more appealing. One way to stabilize an emulsion without a surfactant is to use solid particles, resulting in Pickering emulsions (PEs), where the particles create a robust physical barrier around the droplets by retarding coalescence [2]. Compared to other surfactant-based emulsions, the particles in PEs exhibit significantly higher interfacial adsorption energy, resulting in superior stability [3,4,5]. Biomass-derived polymeric particles, such as polysaccharides (e.g., cellulose, starch) [6], proteins (e.g., Zein-Soybean Polysaccharides Nanoparticles) [7], and fats (e.g., lipid crystals) [8], have been widely used to develop PEs for diverse applications in foods, cosmetics and pharmaceutical industries [9]. In this context, cellulose nanocrystals (CNCs) have significant potential for the development of PEs, given their availability, renewability, sustainability, and low environmental impacts [10]. CNCs have been used as Pickering stabilizers due to their nanoscale dimensions, high surface charge, large surface area, low density and strong van der Waals interactions between the particles [11]. According to Liu et al. [12], CNCs also form a network structure in the emulsion continuous phase that improves the thickening and gelling properties of the PEs by preventing phase separation. Using contrast-matched small-angle neutron scattering, Cherhal et al. [13] showed that CNCs adsorbed as a monolayer at the oil–water interface in Pickering emulsions and inferred an orientation in which the (200) crystalline plane contacted the interface without detectable interfacial deformation. They also form a thick interfacial network barrier around the oil droplets, thereby improving emulsion stability [6].

So far, plant-based CNCs from wood and cotton have been mostly used for PE formulation. Typically, plant-CNCs have a low aspect ratio and high water wettability, which can lead to lower stability of the oil–water interface. Various surface modifications of CNCs have been investigated to reduce their wettability and improve oil–water interfacial adsorption [14,15]. However, the modification process is complex and often relies on significant amounts of chemicals and reagents, many of which may not be food-grade or environmentally sustainable. In this work, we focused on a novel unmodified tunicate-derived cellulose nanocrystal (T-CNC) with long lengths or high aspect ratios, which have not been observed in plant-based CNCs.

Tunicates are marine invertebrates characterized by their unique leather-like epidermis, known as the tunic. They are mainly composed of protein and cellulose, along with other extractives, and are known to be invasive in seawater [16]. The tunic contains highly pure cellulose, tunicin, that can be hydrolyzed using appropriate methods to produce tunicate-derived cellulose nanocrystals (T-CNC) [16,17]. Commercially available wood-based CNCs typically have an aspect ratio of approximately 10–20 and crystallinity of 60–80%, whereas T-CNC possesses a comparatively higher aspect ratio of around 50–100 and crystallinity of around 90% [16,18] along with high surface charge [19]. Pickering particles with a high negative charge can introduce electrostatic repulsion and enhance emulsion stability against high ionic strength [6,11]. In contrast, Pandey et al. showed that acidic de-sulfated CNC with a lower surface charge showed faster interfacial adsorption, thicker interfacial layer and better emulsion stability than alkaline de-sulfated CNC with a higher surface charge [20]. The longer length of T-CNC can also play an essential role in emulsion stability by creating a thick interfacial layer [21] and improving steric hindrance [22,23].

Considering the above perspectives, this study aimed to develop T-CNC-based PEs, with the hypothesis that high-aspect-ratio T-CNC would stabilize oil-in-water (O/W) Pickering emulsions at low CNC concentrations primarily via irreversible interfacial adsorption, driven by their high detachment energy (*E_det_*), and by preventing droplet coalescence at an optimum interfacial coverage. The level of stabilization can also be adjusted by pH due to pH-driven changes in particle wettability. The specific objectives were (a) to characterize the T-CNC extracted from tunicate pulp, (b) to develop O/W PEs using T-CNC at three different concentrations of 0.2%, 0.3%, and 0.4% (*w*/*w*) at two different pH levels (3 and 5), (c) to analyze the effect of ultrasonication on emulsion stability, and (d) to compare the stability of the PEs by characterizing their rheology, droplet size and microstructure after 7-day storage.

## 2. Materials and Methods

### 2.1. Materials

Tunicate pulp, sourced from *Styela clava* tunicate species, was received from Tunistrong Technologies Inc, PEI, Canada and further processed into T-CNC. Canola oil was purchased from the local market in Saskatoon, SK, Canada. Sodium hypochlorite (5%, *w*/*w* solution), sodium hydroxide (NaOH, ≥98% purity), and chromium (III) nitrite (≥98% purity) were purchased from Sigma-Aldrich (Oakville, ON, Canada). Hydrochloric acid was purchased from Fisher Scientific (Toronto, ON, Canada). De-ionized water from Synergy^®^ Water Purification System (MilliporeSigma, Toronto, ON, Canada) with an ionic purity of 18.2 MΩ cm at 25 °C was used for all experiments.

### 2.2. Extraction of Tunicate Cellulose Nanocrystals

The tunicate pulp underwent several pretreatments, and T-CNC was obtained from the pulp using a chromium-based catalytic reaction method, according to Babaei-Ghazvini et al. [24], with some modifications. Briefly, 200 g of the bleached tunicate pulp was treated with chromium catalyst (chromium (III) nitrate) under alkaline conditions using 6 mL 2% (*w*/*w*) NaOH and 300 mL NaOCl. The mixture was heated to a moderate temperature (80–100 °C) with continuous stirring at around 750 rpm for 7–8 h, to facilitate the breakdown of amorphous regions of cellulose by catalyzing oxidative and hydrolytic cleavage of glycosidic bonds. After the reaction, the suspension containing T-CNC was filtered and dispersed in water using a probe sonicator (UP400s, Hielscher Ultrasonics GmbH, Teltow, Germany; 400 W, 80% amplitude) for 2 min, paused for 30 s to avoid overheating, then sonicated for another 3 min. The total solids of the T-CNC dispersion were determined gravimetrically according to AOAC method [25].

### 2.3. Characterization of Tunicate Cellulose Nanocrystals

#### 2.3.1. Morphological Characterization

Transmission electron microscopy (TEM) (HT7700 TEM, Hitachi High-Tech, Tokyo, Japan) was used to examine the morphology of T-CNC. A diluted colloidal suspension of T-CNC (0.001%, *w*/*w*) in water was prepared and ultrasonicated for 1 min to ensure uniform dispersion. The suspension was then cast onto copper grids, stained with 0.5% (*w*/*w*) phosphotungstic acid, and allowed to air-dry before being subjected to TEM analysis. For representative and accurate measurements, only well-isolated nanocrystals exhibiting intact rod-like morphology and uniform staining were selected for analysis. Particles that were bundled, overlapping, folded, or partially obscured were excluded, consistent with established TEM protocols for CNCs. Magnification calibration was verified by allowing the pixel-to-nanometre scale in ImageJ (version 1.54, National Institutes of Health, Bethesda, MD, USA) on macOS. to be adjusted so that measured distances matched the known reference spacing. Micrographs were acquired from multiple non-adjacent grid regions to avoid sampling bias. The length and width of the T-CNC were determined using ImageJ software, with measurements performed on 250 randomly selected nanocrystals from several TEM images to achieve a statistically robust size distribution.

#### 2.3.2. Fourier Transform Infrared (FTIR) Spectral Analysis

T-CNC gel was cast on a Petri dish and air-dried at room temperature. The measurements were carried out using Attenuated Total Reflectance-Fourier Transform Infrared (ATR-FTIR) spectroscopy (Spectrum 3 Tri-Range MIR/NIR/FIR Spectrometer, PerkinElmer, Waltham, MA, USA) in the mid-infrared region (600–4000 cm^−1^). The dried T-CNC films were placed directly on the ATR crystal, and spectra were recorded across the wavenumber with a resolution of 4 cm^−1^ for 32 scans. The raw data were collected and exported to OriginPro version 2020 (OriginLab Corporation, Northampton, MA, USA) for further analysis.

#### 2.3.3. X-Ray Diffraction Measurements

The crystallinity of the T-CNC film (made by drying the T-CNC dispersion at room temperature) was analyzed using an X-ray diffractometer (XRD) (Bruker AXS D8 Advance Instrument, Rigaku Ultima-IV, Woodlands, TX, USA), equipped with a graphite monochromator, adjustable slits, and a scintillation detector. The measurements employed CuKα radiation at a wavelength of 1.54 Å and were conducted in a 2θ range of 10° to 35° at room temperature (~25 °C). The crystallinity index (*CI*) was calculated using the Segal method following Equation (1) [26,27]:

(1) $$CI(\%)=\frac{I_{cr\;}-I_{am}}{I_{cr}}\times 100$$

where $I_{cr\;}$ (*I*_200_) is the maximum intensity of the (200) reflection of cellulose Iβ (near 2θ ≈ 22.5°), and *I_am_* is the minimum intensity between the (110) and (200) reflections (≈18–20°), taken as an estimate of the amorphous contribution.

#### 2.3.4. Zeta Potential of Tunicate Cellulose Nanocrystal Dispersion

The zeta potential of T-CNC was determined using a Zetasizer Nano ZS90 instrument (Malvern Instruments, Malvern, UK). To minimize the multiple scattering effects, five drops of T-CNC dispersion (0.001%, *w*/*v*) were added to 40 mL of pH-adjusted deionized water, with continuous stirring at 300 rpm for 15 min. The pH of the deionized water was adjusted using 0.1 N NaOH and 0.5 N HCl. The samples were transferred to DTS1070 cuvette cells for zeta potential analysis, varying the pH from 2 to 10. Measurements were performed at a refractive index of 1.544 and an absorption index of 1.0 at 25 °C.

#### 2.3.5. Measurement of Contact Angle and Detachment Energy

The static water contact angle of T-CNC film was measured at 25 °C using the sessile drop method with an optical tensiometer (DSA 30 Kruss GmbH, Hamburg, Germany), equipped with a micro syringe and a high-speed digital camera. A straight needle with a 0.5 mm diameter was used to dispense 5.0 μL water droplets (adjusted to pH 3 and 5), forming sessile drops on the T-CNC film. The droplet contours were recorded using a video camera and analyzed with Advanced Drop Image software (version 1.15.0 Kruss GmbH, Hamburg, Germany). The three-phase contact angle (*θ*), defined as the angle through the water droplet, formed at the interface of solid T-CNC, water, and oil phases, is commonly described by the classical Young’s equation [28]:

(2) $$Cos\theta =\left(\gamma_{SO}-\gamma_{SW}\right)/\gamma_{OW}$$

where $\gamma_{SO}$, $\gamma_{SW}\;$ and $\gamma_{OW}$ refer to the interfacial tension between the solid particles and the oil phase, the solid particles and the water phase, and the oil and water phases, respectively.

The detachment energy ($E_{det}$) of spherical particles at the oilwater interface, defined as the energy required to remove the particle from the interface, was calculated using Equation (3) [29,30].

(3) $$E_{det}=\pi r^{2}\gamma_{ow}{\left(1\pm cos\theta \right)}^{2}$$

where *r* is the radius of the particle, $\gamma_{ow}$ is the oil–water interfacial tension, and θ is the contact angle. The ± sign indicates whether the particle detaches into the oil phase (+) or the water phase (−). Based on this equation, Peddireddy and Lisuzzo et al. [31,32] derived the detachment energy for a rod-like particle as:

(4) $$E_{det}=\gamma_{ow}lq\;(1-\;cos\theta )$$

where *l* and *q* are the length and width of the rod-like particle, respectively. Spherical particles have a circular contact line at the interface, so the detachment energy scales with the area ($\pi r^{2}$) and the square of wettability ${\left(1\pm cos\theta \right)}^{2}$. For rod-shaped particles with a linear contact line (along their length), $E_{det}$ scales with the length (*l*) and linearly with wettability (1±cosθ); hence (1− cosθ) was used to calculate the detachment energy of rod-shaped CNC particles, which are hydrophilic and prefer to detach into the aqueous phase.

### 2.4. Fabrication of T-CNC-Based Pickering Emulsion

T-CNC-based PE was developed following the method of Aw et al. [33] with a few modifications. In a preliminary study, a range of T-CNC concentrations (0.1–0.5%, *w*/*w*) was initially prepared by diluting the original T-CNC suspension (from Section 2.2) with de-ionized water, which was then used to develop 20 wt% canola O/W emulsion. Based on visual observations, droplet size, and microscopic assessments, three T-CNC concentrations (0.2–0.4%, *w*/*w*) were selected for further study at two pH levels, 3 and 5. The pH of each T-CNC suspension was first coarsely adjusted using 0.5 N HCl or 0.5 N NaOH, followed by fine adjustments using 0.1 N HCl and 0.1 N NaOH when required. All pH values were verified with a calibrated pH meter prior to emulsification. The selection of the pH range was based on relevant food and cosmetic applications where a neutral to mildly acidic condition is preferred. Preliminary study also revealed that the T-CNC yielded unstable emulsion at pH 7; hence, pH 3 and 5 were selected for the current research.

Emulsions were prepared by mixing canola oil and the T-CNC suspension at 10,000 rpm for 7 min with a high-shear homogenizer (Polytron 2500E, Brinkmann Instruments, Mississauga, ON, Canada). One set of PEs prepared with the high-shear homogenizer was further ultrasonicated using an ultrasonic processor (UP400s, Hielscher Ultrasonics GmbH, Germany) at a frequency of 24 kHz and a power level of 320 W with 80% amplitude for 3 min. The PEs were stored at room temperature (25 °C) in glass vials for 7 days for further characterization.

### 2.5. Characterization of Pickering Emulsions

#### 2.5.1. Droplet Size

Droplet size distribution was measured using a static laser diffraction particle size analyzer (Mastersizer 3000, Malvern Instruments, Montreal, QC, Canada) with a Hydro EV sample handling unit immediately after emulsion preparation and on the 7th day of storage. PE samples were diluted dropwise into 500 mL of pH-adjusted deionized water to reach about 7% obscuration, followed by 30 s of low-energy ultrasonication to gently disperse droplet flocs and CNC–CNC clusters prior to laser diffraction analysis. The relative refractive index, calculated as the ratio of canola oil (1.456) to the dispersion medium (1.33), was 1.095. The average droplet sizes were calculated as volume-average diameter (*D_43_*).

#### 2.5.2. Zeta Potential

The zeta potential of the T-CNC-stabilized PE samples was measured with the same instrument used for the zeta potential of T-CNC (Section 2.3.4). The PE samples were diluted by adding three drops of the sample to 40 mL of pH-adjusted deionized water, with continuous stirring at 300 rpm for 30 min to ensure uniform dispersion. The diluted samples were placed into DTS1070 cells, transferred to the instrument to determine the electrophoretic mobility of the droplets, which was used to calculate the zeta (ζ) potential.

#### 2.5.3. Rheology

The viscosity and viscoelasticity of freshly prepared and seven-day-stored emulsions were measured using a rheometer (AR G2, TA Instruments, Montreal, QC, Canada) equipped with a 40 mm cross-hatched parallel plate geometry. Samples were placed on the lower Peltier plate with a disposable pipette. Viscosity was determined by applying rotational shear between the parallel plates at 25 °C, with a 1000 μm gap, across a shear rate range from 0.1 s^−1^ to 1000 s^−1^. The Herschel–Bulkley parameters (yield stress, consistency coefficient, and flow behaviour index) were extracted by fitting the shear stress-shear rate data to Equation (5) using OriginPro 2020 (OriginLab Corporation, Northampton, MA, USA). To assess viscoelasticity, oscillatory strain and frequency sweeps were performed. Strain sweep measurements for storage (G′) and loss moduli (G″) of the emulsions were conducted over a strain range of 0.01–1000% at a constant frequency of 1 Hz (6.28 rad/s) to identify the linear viscoelastic region (LVR). Based on the obtained data, a constant strain amplitude of 0.1% within the LVR was selected for subsequent frequency sweep measurements from 0.01 Hz to 100 Hz (1 to 100 rad/s). All the rheology data were recorded and the crossover modulus and yield strain was calculated from the strain sweep measurements using TRIOS software (version 4.5.0.42498, TA Instruments, Montreal, QC, Canada).

#### 2.5.4. Microstructure

A polarized light microscope (BA310POL; Motic, Inc., Richmond, BC, Canada) operated in bright-field mode and fitted with a digital camera (Moticam 3; Motic, Richmond, BC, Canada) was used to image the PEs. An adequate amount of sample was placed on a glass slide, covered with a cover slip, and the images were captured at 40× and 100× magnifications using Motic Live Imaging Module (Motic, Inc., Richmond, BC, Canada).

### 2.6. Statistical Analysis

All experiments were performed in triplicate, and results are reported as mean ± standard deviation. A three-way analysis of variance (ANOVA) was performed to determine the statistical significance of T-CNC concentration, pH and storage on the characteristics of the PEs. The effect of ultrasonication in emulsion preparation was examined in a separate two-way ANOVA with factors of emulsification process (high shear vs. high shear and ultrasonication) and storage time. Statistical analyses were performed using OriginPro version 2020 (Origin Lab Corporation, Northampton, MA, USA) with Tukey post hoc test and a 95% confidence interval.

## 3. Result and Discussion

### 3.1. T-CNC Characterization

#### 3.1.1. Morphology

**Figure 1:**
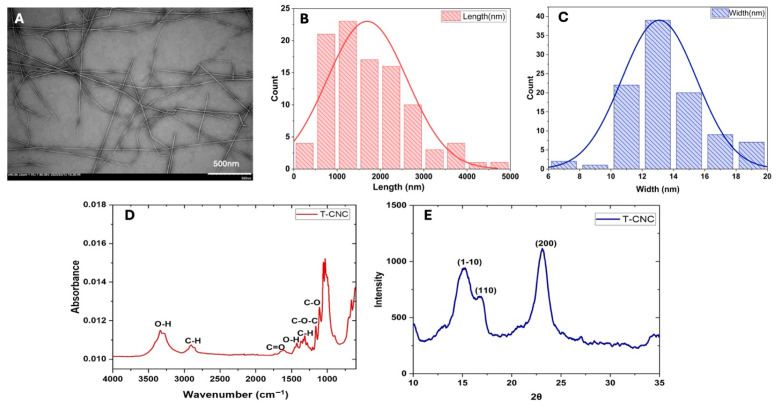
Characteristics of tunicate cellulose nanocrystals (T-CNC): (**A**) TEM image, (**B**,**C**) particle length and width distribution calculated from image analysis, (**D**) FTIR spectrum, and (**E**) XRD spectrum.

TEM images (Figure 1A) revealed a long rod-shaped, fibre-like nature of the T-CNC particles in the dispersion. The histograms of the distribution of the length and width of the particles (calculated using image analysis) (Figure 1B,C) showed that the rod-shaped T-CNC particles had a mean length of 1694 ± 925 nm, a width of 13 ± 3 nm and an average aspect ratio (AR) of 122 ± 45. The TEM image also revealed the side-axis aggregation of T-CNC, forming a structure with greater directional variation due to the uneven alignment of CNC along their length during the aggregation process. The high aspect ratio of T-CNCs aligns with previous reports for sulfuric acid hydrolysis isolation-derived T-CNCs from *Styela clava* (length ≈ 1567 nm, AR ≈ 90) and *Ciona intestinalis* (length ≈ 1374 nm, AR ≈ 80), indicating broadly similar dimensions across species [19]. The oxidized T-CNC in the present work shows a higher aspect ratio than reported by Dunlop et al. [19], and Babaei-Ghazvini & Acharya [17] (AR ≈ 80), which could be attributed to the different extraction methods (sulfuric acid hydrolysis vs. metal-assisted oxidation).

#### 3.1.2. Fourier Transform Infrared (FTIR) Spectroscopy

The FTIR spectra of the T-CNC (Figure 1D) showed different peaks representing different functional groups, almost similar to the sulfuric acid hydrolyzed T-CNC spectra [19]. The broad peaks around 3400 cm^−1^ correspond to the -OH stretching vibration of hydrogen bonds, confirming the strong water affinity of T-CNC. This might be attributed to its polysaccharide structure, which contains abundant hydroxyl groups [34]. The peaks around 2900 cm^−1^ and 1400 cm^−1^ suggest the presence of alkyl groups (methyl and methylene), which are part of the cellulose backbone [35]. The peak around 1627 cm^−1^ indicates the presence of carbonyl (C=O) groups, which could be due to oxidation during the extraction and bleaching process in the presence of NaOH [36]. The peaks between 1050 and 1150 cm^−1^ are characteristic of ether linkages, attributed to C-O-C stretching vibrations of the glycosidic linkages in cellulose [36].

#### 3.1.3. X-Ray Diffractometry

The XRD pattern (Figure 1E) shows characteristic peaks for T-CNC and identifies the crystalline structure of the nanocellulose. The reflection at 2θ ≈ 15° corresponds to the (1–10) plane, which reflects the side-by-side packing of cellulose chains. The peak at 2θ ≈ 16.5° arises from the (110) plane, providing additional information on lateral chain arrangement. The most intense peak at 2θ ≈ 22.5° corresponds to the (200) plane, representing the main crystalline stacking direction and overall structural order of cellulose Iβ. This peak pattern of XRD is similar to the sulfuric acid-hydrolyzed T-CNC studied by Dunlop et al. [19]. The 2θ values and the peak intensity of T-CNC indicate the retention of the crystalline structure of cellulose after their extraction. The CI of the T-CNC, calculated using Equation (1), was 87.6%, which is significantly higher than that of plant-based CNC, such as cotton (80%) [37] and oat husk (57%) [38] and almost similar to fungal CNC (84%) [35]. The results suggested that much of the amorphous region of T-CNC was removed during the oxidation reaction.

#### 3.1.4. Surface Charge

**Figure 2:**
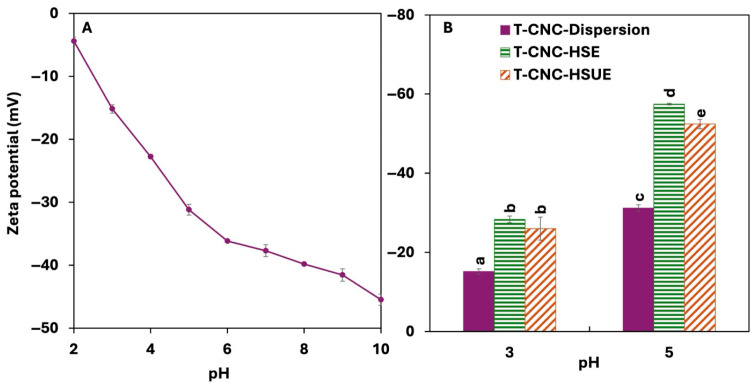
(**A**) Surface charge of tunicate cellulose nanocrystals (T-CNC) dispersion as a function of pH from 2 to 10. (**B**) Surface charge of T-CNC dispersions and T-CNC-stabilized Pickering emulsion droplets at pH 3 and pH 5, prepared by high-shear homogenization (HSE) and by high-shear homogenization plus ultrasonication (HSUE). Bars show means ± SD (n = 3). A two-way ANOVA spanning both dispersions and emulsions was applied with fixed factors of CNC condition (dispersion, T-CNC-HSE, T-CNC-HSUE) and pH, and their interaction was tested. Bars that do not share a letter differ significantly (*p* < 0.05).

The surface charge of T-CNCs increased with pH, from −4.42 mV at pH 2 to −45.5 mV at 10 (Figure 2A). T-CNCs possess a high surface charge of −37.7 mV at their native pH (pH 7), which likely arises from the presence of surface carboxylate groups (-COO^−^) generated during the oxidation process. Unlike cationic polysaccharides, CNCs do not contain intrinsic positively charged groups [39]. Their strong anionic nature is primarily due to the surface carboxylates (from oxidation) [40]. The rise in pH deprotonates surface carboxylate groups, resulting in an increase in negative charge [41]. Due to their high surface charge, T-CNC remained well-dispersed in colloidal suspensions, as interparticle electrostatic repulsion prevents aggregation. Sulfuric acid hydrolyzed T-CNC also showed a similar high surface charge of −41 mV at pH 10 [16]. The surface charge dropped as the pH was lowered to strongly acidic conditions due to protonation of the carboxylate groups. A study on ammonium persulfate oxidation (APS-CNC) of Eucalyptus-CNC showed a higher surface charge at −33.3 mV at pH 7 and a lower surface charge at −12.6 mV at pH 2 [42]. Other studies on APS oxidation to generate CNC reported surface charge of −45.4 to −52.2 mV at pH 6.5 with the variation in oxidation treatment [43], while TEMPO oxidation also resulted in a surface charge of −49.1 mV at pH 9.5 [44], both in agreement with the present study.

#### 3.1.5. Contact Angle and Detachment Energy

A key performance evaluation parameter for solid particles in stabilizing PEs is their three-phase contact angle at the oil–water–particle interface [45]. When the contact angle approaches 90°, the interfacial adsorption capacity of the particles increases, promoting the formation of a stable emulsion. Particles with θ<90°, are more hydrophilic, favouring O/W emulsions, while those with θ>90°, are more lipophilic, favouring W/O emulsions [46]. The average contact angle of T-CNC measured in this study was 30.2 ± 2.4° at pH 3, decreasing to 22.0 ± 1.6° at pH 5, indicating its high hydrophilicity. These values are consistent with those reported in the literature. For example, a contact angle of 42° at pH 7 and 21° at pH 8 was reported by Wong et al. [47] and D’Acierno & Capron. [48], respectively.

The surface displacement energy (*E_det_*) of the T-CNC particles was calculated using Equation (4) from their contact angle and rod-shaped geometry Table 1. *E_det_* represents the energy required to detach a particle from the oil–water interface, and thus a higher *E_det_* indicates stronger particle anchoring, which contributes to improved emulsion stability [9,46]. In this study, *E_det_* was 16,206 kT (6.67 × 10^−17^ J) at pH 3 and 8808 kT (3.62 × 10^−17^ J) at pH 5. The higher *E_det_* at pH 3 reflects reduced wettability of T-CNCs under acidic conditions, promoting anchoring at the interface. In contrast, the lower *E_det_* at pH 5 corresponds to increased wettability, weakening interfacial anchoring. Zhao et al. [49] showed that higher carboxylation of TEMPO-oxidized cellulose nanofibres reduced contact angle (51.5° → 37.7°) and *E_det_* (21.69 → 5.45 × 10^−18^ J), due to an increased surface carboxyl group, enhancing negative charge and hydrophilicity. Similarly, in the present work, metal-oxidized CNCs contain carbonyl and carboxyl groups that deprotonate at higher pH, increasing negative charge, lowering the contact angle, and reducing *E_det_*. Thus, both chemical carboxylation and pH-induced ionization alter wettability through surface charge regulation, ultimately controlling particle detachment energy and interfacial stability.

In addition to surface chemistry, *E_det_* is also influenced by particle size and geometry. Larger or elongated particles provide stronger interfacial anchoring than smaller ones. Lisuzzo et al. [31] found a similar result for rod-shaped nanotubes with a contact angle of 25.6° and an *E_det_* of 3.45 × 10^4^ kT. While spherical particles generally yield higher *E_det_* due to a squared dependence on (1 ± cosθ), rods with lengths far exceeding their width can require even greater energy to detach. Moreover, the ability of rods to form end-to-end linkages at the interface enhances interfacial coverage and further increases detachment energy, thereby prolonging emulsion stability. Considering both properties together, these findings demonstrate that both surface chemistry (pH-dependent ionization and functionalization) and particle geometry (size and aspect ratio) can act synergistically to determine the detachment energy and overall stability of CNC-based Pickering emulsions.

### 3.2. Characterization of T-CNC-Stabilized PEs

#### 3.2.1. Droplet Size of T-CNC-Stabilized PEs

**Figure 3:**
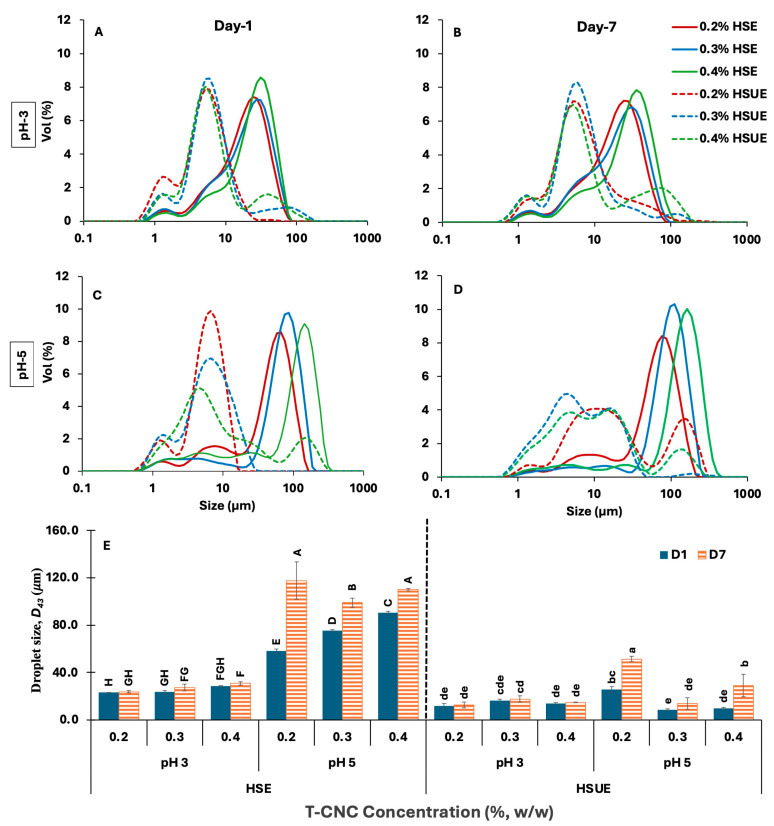
(**A**–**D**) Droplet size distribution of CNC-based Pickering emulsions stabilized with different concentrations (0.2–0.4%, *w*/*w*) of tunicate CNCs (T-CNC). (**A**) pH 3 day 1, (**B**) pH 3 day 7, and (**C**) pH 5 day 1, (**D**) pH 5 day 7. HSE: high-shear homogenization-based emulsions (solid lines). HSUE: high-shear homogenization plus ultrasonication-based emulsions (dashed lines). (**E**) Volume average droplet diameter (D43) of all emulsions as a function of T-CNC concentration (0.2–0.4%, *w*/*w*), pH (3, 5), emulsification methods (HSE, HSUE), and storage time (day 1 and 7). Bars that do not share a letter differ significantly (*p* < 0.05). For HSE and HSUE, groupings from a three-way ANOVA (pH × concentration × storage) are shown with uppercase letters (A–H) and lowercase letters (a–e), respectively.

The droplet-size distributions were multimodal with large peaks at droplet diameters > 1 µm under all conditions (Figure 3A–D). A minor peak near 1 µm likely originated from CNC clusters and/or very small droplets under both processing conditions (HSE and HSUE). Increasing T-CNC from 0.2% to 0.4% (*w*/*w*) shifted the distributions toward larger sizes and significantly increased the volume-average diameter *D_43_* (Figure 3E and Table S1) at both pH values: at pH 3 from 23.1 to 28.4 µm, and at pH 5 from 57.9 to 90.5 µm. This trend contrasts with many reports on plant-derived CNCs (typically 100–300 nm long), in which higher CNC concentrations, typically 1–3% (*w*/*w*), reduced droplet size until an optimal coverage was reached [22,33,51]. Because those systems rely on substantially higher particle loadings, direct numerical comparison of droplet size is inappropriate. At the low concentrations used here (0.2–0.4%, *w*/*w*), the more viscous continuous phase and stronger T-CNC aggregation likely reduce interfacial coverage efficiency and resulting in larger droplets. This behaviour appears specific to long, oxidized T-CNCs, whose strong interactions differ markedly from shorter, highly charged plant CNCs reported previously [6,52,53].

A similar increasing trend of droplet size with increased CNC concentration was not observed when ultrasonication was used (HSUE). The effect of pH on droplet size depended strongly on the emulsification method. With HSE, droplets were consistently larger (*p* < 0.05) at pH 5 than at pH 3, consistent with lower detachment energy and weaker interfacial anchoring at the higher pH. With HSUE, pH effects became concentration-dependent; at 0.2% (*w*/*w*), the smallest droplets formed at pH 3 (11.6 vs. 25.7 μm), whereas at 0.3–0.4% (*w*/*w*), smaller droplets formed at pH 5 (8.5–9.67 μm vs. 16.20–13.97 μm). Similar pH-concentration interactions have been reported for plant CNC systems, although the direction of the pH effect is not always consistent across CNC sources. Highly sulfated plant CNCs often show larger droplets at low pH due to aggregation and reduced effective particle availability [52,54], whereas our low-charge T-CNCs display improved coverage and anchoring at pH 3. These differences underscore that pH-dependent wettability and anchoring behaviour are strongly CNC-source-dependent.

Seven-day storage broadened the distributions and increased *D_43_* for all samples from Day 1 to Day 7 (*p* < 0.05), reflecting droplet coalescence. The increase was consistently smaller for HSUE than for HSE, indicating greater resistance to coalescence after sonication. Although the extent of coarsening in our dilute T-CNC systems is larger than typically reported for concentrated plant-CNC emulsions (>1%, *w*/*w*), the qualitative trend, reduced growth when interfacial coverage is improved, is consistent with earlier work [54,55]. At similar concentration and processing conditions, pH 3 preserved *D_43_* more effectively than pH 5, consistent with higher detachment energy and lower surface charge at low pH. Again, this differs from some plant-CNC studies where low pH induces particle aggregation and leads to poorer stability [52,55].

Finally, the emulsification methods had the strongest effect on mean droplet size. Relative to HSE, HSUE shifted the distributions to smaller diameters, narrowed the peaks, and reduced D_43_ at every condition (*p* < 0.05). The HSE to HSUE reductions were 50.0%, 31.4%, and 50.8% at pH 3 and 55.7%, 88.6%, and 89.3% at pH 5 for 0.2%, 0.3%, and 0.4% (*w*/*w*) T-CNC concentrations, respectively. Although the absolute droplet sizes remain larger than those commonly reported for plant CNC systems stabilized at much higher CNC concentrations (1–3%, *w*/*w*), the mechanistic improvements enhanced CNC de-aggregation and droplet breakup, mirroring the same qualitative behaviours described for smaller CNCs [56,57].

When compared with other CNC-stabilized O/W emulsions containing similar oil content (≈20 wt%), our HSUE emulsions fall into the expected micron-scale range but at far lower CNC concentrations. Varanasi et al. [51] reported 1–4 µm droplets at 1–3% (*w*/*w*) CNC using probe ultrasonication; Bai et al. [22] obtained 1–3 µm droplets at 0.75% (*w*/*w*) CNC using microfluidization; and Perrin et al. [53] showed a decrease from ~17 µm to ~2 µm when CNC concentration increased from 0.1% to 1.5% (*w*/*w*). In contrast, our T-CNC emulsions achieve long-term stability at only 0.2–0.4% (*w*/*w*). CNC, despite having larger absolute droplet sizes, the droplet-to-particle size ratio (≈10–20×) remains consistent with the requirement that droplets be at least an order of magnitude larger than CNC length, although this ratio is numerically lower than that reported for shorter plant CNCs (≈40–60×) [22,55]. These distinctions reflect the unique high-aspect-ratio, non-sulfated characteristics of T-CNCs, which permit stable emulsions at much lower particle loadings than typically needed with plant-derived CNCs.

Overall, the combined effects of concentration (coverage efficiency), pH (wettability and anchoring strength), storage (coarsening), and sonication (breakup and de-aggregation) can explain the observed change in mean droplet sizes and their distributions. While the absolute values differ from most plant-CNC studies, the mechanistic trends are consistent, and the ability of high-aspect-ratio T-CNCs to stabilize emulsions at a significantly lower particle concentration represents a central finding of this work.

#### 3.2.2. Droplet Surface Charge

The surface charge of droplets varied with pH and emulsification method but was unaffected by T-CNC concentration or storage (Figure 2B). For HSE, surface charge increased from −28.4 mV at pH 3 to −57.5 mV at pH 5, following the same trend as T-CNC dispersions. This agrees with Shin & Hyun [58], who showed that cotton-derived sulphated CNC-stabilized droplets also exhibited higher charges with increasing pH. In the present case, droplet charges were consistently higher than the particles, likely due to denser CNC adsorption at the oil–water interface. Droplet charge for ultrasonicated emulsions (HSUE) showed slightly less negative values than the HSE. This contrasts with Meirelles et al. [57], who reported higher surface charges in CNC suspensions following ultrasound treatment. The authors evaluated CNCs in bulk suspension, where ultrasound mainly breaks aggregates and increases dispersion quality. In our case, surface charge was measured on CNC-coated droplets, where probe ultrasonication can induce droplet breakup, thereby increasing interfacial area and can reduce the effective surface coverage of adsorbed TCNCs, leading to a reduction in droplet charge.

#### 3.2.3. Flow Behaviour of T-CNC-Stabilized PEs

**Figure 4:**
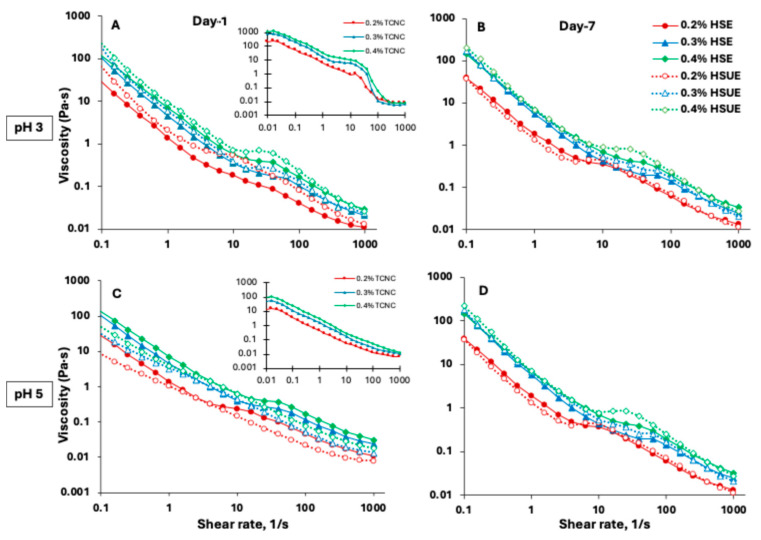
Viscosity as a function of shear rate for T-CNC-based Pickering emulsions (PEs) prepared with high-shear homogenization (HSE, solid lines and solid symbols) and high-shear homogenization plus ultrasonication (HSUE, dashed lines and open symbols). (**A**) pH 3 day 1, (**B**) pH 3 day 7, and (**C**) pH 5 day 1, (**D**) pH 5 day 7. Emulsions were stabilized with 0.2% (red), 0.3% (blue), and 0.4% (green), (*w*/*w*) T-CNC dispersions. Insets in (**A**,**C**) show flow curves of the corresponding T-CNC dispersions (0.2%, 0.3%, 0.4%, (*w*/*w*) at the same pH) before emulsification.

Viscosity analysis provides key insights into emulsion stability and flow behaviour, which are critical for food, cosmetic, and pharmaceutical applications. Viscosity of T-CNC dispersions (Figure 4A,C, inset) showed a strong dependence on shear rate, pH and concentration. Below 5% shear rate, shear-thinning aligned T-CNCs along the shear. At an intermediate shear regime (below 50% shear rate), all particles were oriented along the shear. A second shear-thinning regime occurred at higher shear rates (up to 100%), followed by a very high-shear plateau beyond 100% shear rate (Figure 4A, inset). Typically, anisotropic rod-shaped particles like CNC show a three-stage shear-thinning behaviour with a high-shear plateau in dilute T-CNC dispersions, which could result from the complete breakdown of particle–particle interactions. Moreover, the viscosity of pH 3 T-CNC dispersions at 0.4% (*w*/*w*) reached about 1200 Pa·s at 0.01% shear rate, significantly higher than previous reports of much smaller size CNC dispersions at higher concentrations [59,60]. However, at pH 5, the viscosity significantly dropped to about 100 Pa·s at 0.01% shear rate (Figure 4C, inset). The stepwise shear-thinning behaviour was absent at pH 5, which could be attributed to the higher surface charge of the particles, leading to a lack of interparticle interactions [61].

The flow behaviour of the T-CNC-stabilized PEs was also dependent on the shear rate, pH and particle concentration. At pH 3, the initial low-shear-rate viscosity of the PEs was significantly lower than that of the corresponding dispersions (Figure 4A vs. inset), which could be attributed to oil droplets acting as inactive fillers in the T-CNC dispersions, breaking the T-CNC particle network. Also, due to interfacial adsorption, there were fewer T-CNC particles available in the continuous phase, leading to an overall lowering of PE viscosity. Similar findings have been reported for wood-derived CNCs, where suspensions (1–5%, *w*/*w*) displayed high viscosity, but emulsions prepared from the same suspensions showed reduced viscosity because a portion of CNCs adsorbs at oil–water interfaces rather than contributing to bulk phase viscosity [53]. At pH 5, no such change in viscosity was observed, and the T-CNC dispersion and the PE showed similar viscosity (Figure 4C). As a function of shear rate, all T-CNC-stabilized PEs (Figure 4A–D) showed a three-stage shear thinning behaviour, with a low and high-shear thinning interrupted by a brief mid-shear plateau, which could be attributed to the shear alignment of oil droplets and T-CNC particles in the continuous phase as described above. Miao et al. developed wood-pulp CNC-stabilized highly concentrated PEs (65–85% oil), which exhibited one-step shear-thinning behaviour [11]. In the presence case, a much larger size of the T-CNC and lower oil concentration in the PEs led to a three-step shear thinning typically observed for concentrated CNC dispersions [59].

##### Modelling Flow Behaviour of T-CNC-Stabilized PEs

To better compare the effects of T-CNC concentration, pH, processing method, and storage time on emulsion flow behaviour, the viscosity data were fitted to the Herschel–Bulkley (HB) model over a shear rate range of 0.1–1000 s^−1^, yielding R^2^ values between 0.935 and 0.991, which provides a relationship between shear stress (τ) and shear rate (γ) (Equation (5)), where *n* is the flow behaviour index, K is the consistency coefficient and $\tau_{ο}$ is the yield stress. The model fitting process ignored the three-step shear thinning behaviour.


(5) $$\tau =\tau_{ο+K{\overset{\cdot }{\gamma }}^{n}}$$

**Figure 5:**
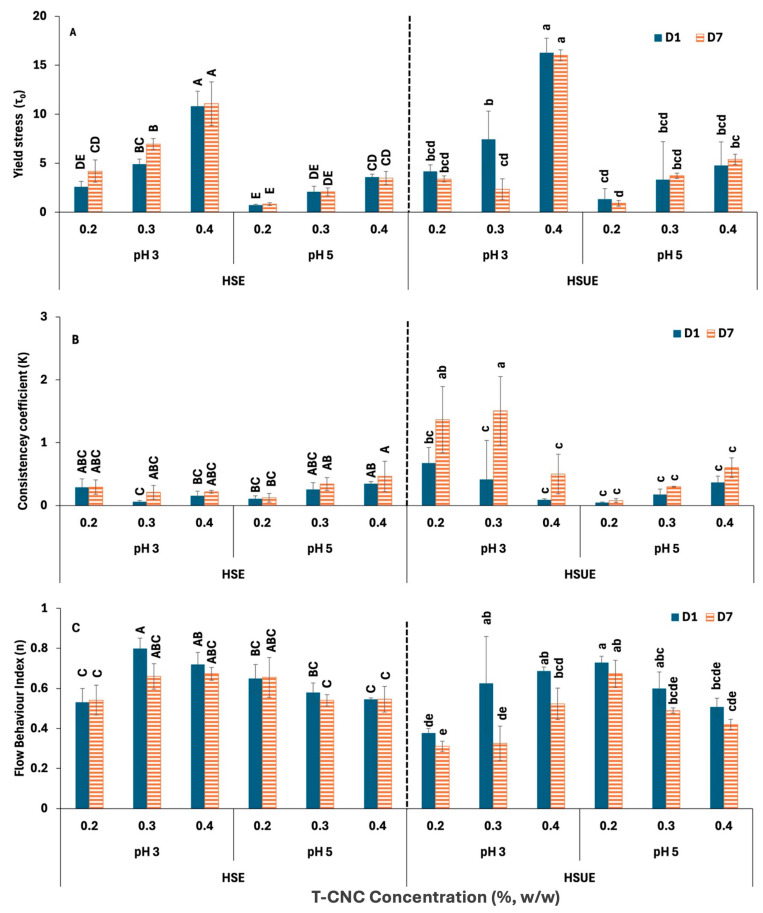
Herschel–Bulkley Model fitting data for T-CNC-based Pickering emulsions at pH 3 and 5 as functions of storage (Day 1, Day 7) and concentration (0.2–0.4%, *w*/*w*). (**A**) Yield stress ($\tau_{ο}$); (**B**) Consistency coefficients (K); (**C**) Flow behaviour index (*n*). HSE: high-shear homogenization-based emulsions, HSUE: high-shear homogenization plus ultrasonication-based emulsions. Bars that do not share a letter differ significantly (*p* < 0.05). For HSE and HSUE, groupings from a three-way ANOVA (pH × concentration × storage) are shown with uppercase letters (A–E) and lowercase letters (a–e), respectively. Data show means ± SD (n = 3).

Figure 5 presents the HB model parameters for all the T-CNC-stabilized PEs. Yield stress (τ_0_) (Figure 5A) indicates the internal network strength of the PEs that resist flow initiation. At pH 3, 0.4% (*w*/*w*) HSE and HSUE showed significantly (*p* < 0.05) higher yield stress than 0.2% and 0.3% (*w*/*w*), confirming stronger particle–droplet networks at higher CNC concentration. However, at pH 5, the increase in yield stress with CNC concentration was much less for both emulsification methods. For the same concentration, emulsions at pH 3 consistently exhibited higher yield stress than those at pH 5. Storage (day 1 vs. day 7) produced no significant changes, except a decrease at 0.3% (*w*/*w*) T-CNC HSUE. Sonication further increased yield stress at pH 3, particular at 0.4% (*w*/*w*) T-CNC-PE, due to smaller droplets and improved interfacial particle coverage. Consistency coefficient (K) (Figure 5B) reflects apparent viscosity beyond the yield point at a shear rate of 1 s^−1^. For HSE, CNC concentration did not significantly affect K. In HSUE at pH 3, 0.2–0.3% (*w*/*w*) T-CNC-PE gave significantly (*p* < 0.05) higher K than 0.4% (*w*/*w*) T-CNC-PE, showing moderate loadings were optimal. At pH 5, no concentration effect was observed. K values were not significantly different across all the HSE emulsions. While at the same concentration level, the pH 3 emulsion showed higher K values than the pH 5. With storage time, K values for HSE remained unchanged, while HSUE at pH 3 showed significant increases in K at 0.2–0.3% (*w*/*w*) T-CNC-PE after 7 days. The flow behaviour index (*n*) represents the degree of pseudoplasticity of the emulsions. From Figure 5C, it can be seen that all emulsions demonstrated non-Newtonian, shear-thinning behaviour (n < 1), characteristics of structured colloidal systems [62]. For HSE, *n* ranged from 0.5 to 0.8 with only minor differences. For HSUE at pH 3, *n* range increased from 0.4 to 0.7 with T-CNC concentration, while at pH 5, concentration effects were minimal. Storage for 7 days did not affect *n* in general, but in HSUE, at 0.3–0.4% (*w*/*w*) CNC at pH 3, *n* dropped with storage (more shear-thinning). Comparing the effect of sonication, *n* dropped at pH 3 (more structured system), but no significant effect was observed at pH 5.

#### 3.2.4. Viscoelasticity of T-CNC-Stabilized PEs

**Figure 6:**
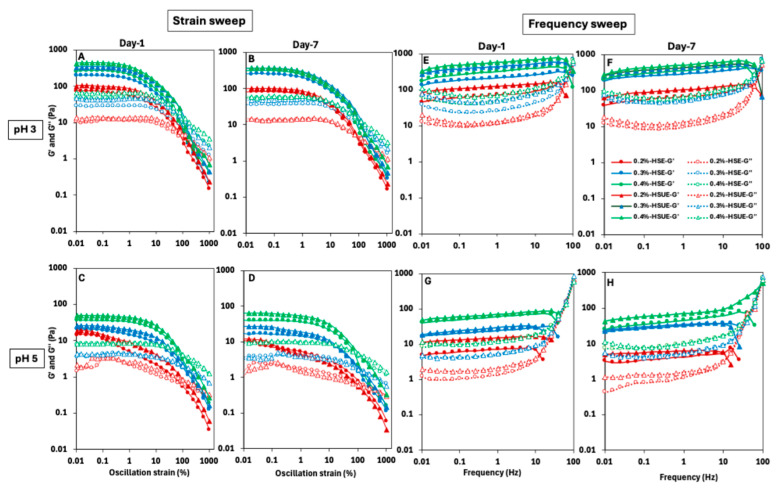
Viscoelastic behaviour of T-CNC-stabilized Pickering emulsions at pH 3 (top row) and pH 5 (bottom row). (**A**–**D**) strain sweep and (**E**–**H**) frequency sweep results. Emulsions were prepared by high-shear homogenization (HSE, circles) or by HSE followed by ultrasonication (HSUE, triangles) and evaluated on Day 1 and Day 7. Storage moduli (G′, closed symbols) and loss moduli (G″, open symbols) are plotted for 0.2%, 0.3%, and 0.4% (*w*/*w*) T-CNC, coded as red, blue, and green, respectively.

The viscoelastic behaviour of the PEs as influenced by T-CNC concentration, pH, emulsification methods, and storage time was determined, and the results are shown in Figure 6A–D for strain-sweep and Figure 6E–H for frequency-sweep. Overall, the storage modulus (G′) was higher than the loss modulus (G″) for all PEs in the low-strain and low-frequency zones, indicating gel-like behaviour of the samples, though the magnitude of viscoelastic moduli within the LVR varied, indicating differences in network strength and structural integrity. During the strain sweep, a linear viscoelastic region (LVR) appeared in most PEs where G′ remained constant. The LVR was shorter at pH 3 than at pH 5. Beyond ~10% strain, a pronounced drop in G′ was observed, indicating irreversible structural breakdown due to yield strain as the droplet and T-CNC particle network collapsed and possibly droplet coalescence occurred, consistent with previous reports on CNC-stabilized emulsions [63,64]. The T-CNC concentrations showed a significant effect, and both G′ and G″ values were comparatively higher at high concentrations. The G′ values at pH 3 consistently exceeded 100 Pa, except the PEs stabilized with 0.2% (*w*/*w*) T-CNC, indicating strong gel formation, while pH 5 PEs remained significantly below 100 Pa, suggesting weaker network structures (Figure 6A–D). At 0.2% (*w*/*w*) T-CNC, pH 5 PEs behaved as weak gels, with no LVR. While increasing T-CNC concentration strengthens the networks at both pH levels, PEs at pH 3 consistently formed stronger gels than those at pH 5 across all concentrations. Pandey et al. [20] also reported higher G′ values for acidic de-sulfated CNCs with lower surface charge, which is comparable to the low-pH condition in our study. This behaviour was attributed to stronger CNC–CNC interactions arising from enhanced van der Waals and hydrogen bonding at the droplet interface, in contrast to alkaline de-sulfated CNCs with higher surface charge that exhibited weaker interparticle associations [20]. The use of ultrasonication showed a higher G′ and G″ values for all fresh PEs due to a reduction in droplet size and improved emulsion stability. A similar trend in the strain and frequency sweep profile was also evident in the T-CNC dispersion (Figure S1), although the values were lower than the corresponding T-CNC-based PEs.

In case of frequency sweep tests, significantly higher G′ was observed than G″ across all the emulsions, until about 10 Hz, indicating elastic gels (Figure 6E–H). A frequency-dependent linear increasing trend of G′ was also noticed for most of the PEs in the range of 0.01 to 10 Hz of frequency, followed by a sudden drop, while the G″ initially decreased at a low frequency and then increased near and beyond the crossover. The gradual increase in G′ with frequency could be ascribed to the close interactions of the droplets and T-CNC network up to a critical packing, beyond which it starts to decline due to the increased difficulty in droplet rearrangement at a higher frequency, and ultimate breakdown of the structure [64,65]. At low frequencies, the droplets and T-CNC networks have time to respond to the applied frequency, leading to a minimal energy loss and lowering of G″. In contrast, at high frequencies, the structure cannot keep up with rapid changes, more energy is lost, leading to an increase in G″, and the dominating viscous behaviour. This frequency-dependent transition from elastic to viscous response is characteristic of viscoelastic gel systems [65,66]. Similarly to oscillatory strain sweep, increasing T-CNC concentrations also resulted in higher elastic behaviour across all PEs in the frequency sweep. Also, a higher elastic behaviour was noticed at pH 3 than at pH 5. In the case of storage effect, at pH 3, no change in elastic behaviour was observed after seven days, whereas a visibly lower G′ and G″ was noticed at pH 5 after seven days compared to the fresh PEs.

##### Comparison of Storage Moduli, Tan δ and Viscoelastic Crossover Strain of T-CNC-Stabilized PEs

**Figure 7:**
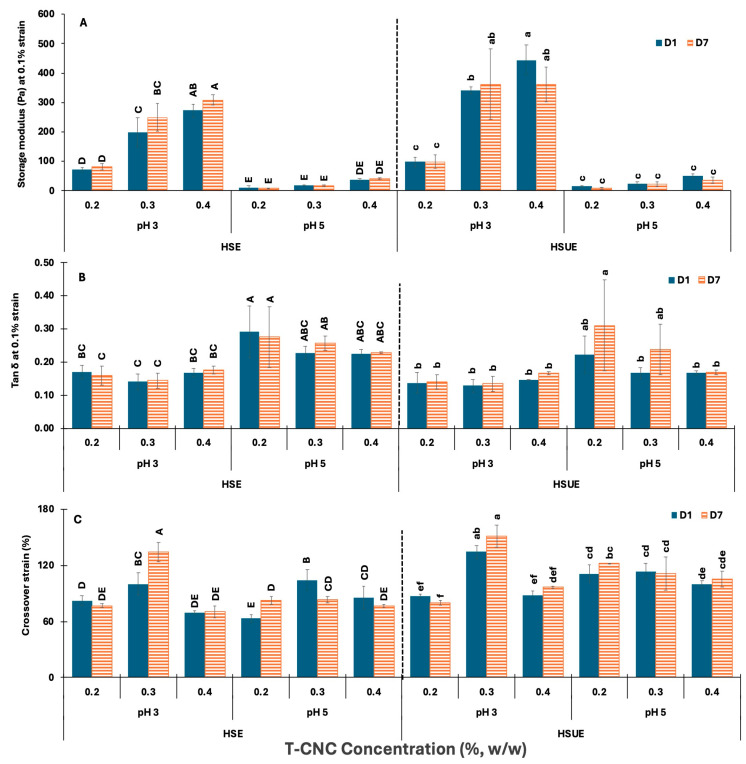
Viscoelastic properties of T-CNC-based Pickering emulsions at pH 3 and 5 as functions of storage (Day 1, Day 7) and concentration (0.2–0.4%, *w*/*w*). (**A**) Storage moduli *G*′ at 0.1% strain; (**B**) Loss tangent (tan δ) at 0.1% strain; (**C**) Crossover strain (%) at *G*′ = *G*″. HSE: high-shear homogenization-based emulsions, HSUE: high-shear homogenization plus ultrasonication-based emulsions. Bars that do not share a letter differ significantly (*p* < 0.05). HSE and HSUE, groupings from a three-way ANOVA (pH × concentration × storage) are shown with uppercase letters (A–E) and lowercase letters (a–f), respectively. Data show means ± SD (n = 3).

The elastic strength and rigidity of the various emulsions were estimated by comparing their storage moduli in the LVR (at 0.1% strain), tan δ values (G″/G′) at 0.1% strain, and G′ and G″ crossover strain at the point of structural breakdown from elastic to viscous behaviour (Figure 7). In Figure 7A, G′ increased with CNC concentration in both HSE and HSUE at pH 3. At pH 5, G′ values were very low for all concentrations, showing very minimal increase with concentration in both emulsification methods. For the same CNC loading, G′ at pH 3 was significantly (*p* < 0.05) higher than at pH 5, reflecting tighter network formation due to reduced electrostatic repulsion among the T-CNC-coated droplets. G′ was stable across storage times (Day 1 vs. Day 7) for the same CNC concentration at both pH levels. Sonication further increases G′ at pH 3, especially at 0.3–0.4% (*w*/*w*), confirming that ultrasonication enhances the formation of elastic, solid-like networks. This can be attributed to finer droplet size distribution and improved dispersion, which allow more efficient adsorption of CNC at the interfaces and stronger interparticle bridging. Additionally, ultrasonication disperses CNC aggregates more efficiently, enhancing particle–particle connectivity in the continuous phase. Together, these effects result in more elastic, gel-like emulsions compared to those produced by HSE alone, consistent with previous observations in plant-derived CNC emulsions where ultrasonication enhanced interfacial packing and viscoelastic strength [66,67].

The tan δ (G″/G′) values at a fixed strain of 0.1% were used to assess the elastic–viscous balance of emulsions (Figure 7B). In HSE, tan δ showed small variation at pH 3 (<0.20) with CNC concentration, and the pH 3 values remained lower than pH 5 (0.2–30.3). Similarly, in HSUE, tan δ was also consistently <0.20 at pH 3, confirming elastic-dominant networks, while at pH 5, values exceeded 0.20, indicating greater viscous contribution. Storage caused no significant (*p* ≥ 0.05) changes.

The crossover strain (Figure 7C) indicates the transition from elastic to viscous behaviour. At pH 3, both HSE and HSUE showed the highest values at 0.3% (*w*/*w*) T-CNC-PE, indicating greater structural flexibility, while 0.4% (*w*/*w*) dropped slightly, suggesting stiffer but more brittle networks. By contrast, pH 5 emulsions formed weaker but relatively more deformable networks, as their crossover strain was less affected by CNC concentration in both emulsification conditions. Storage had little effect on crossover strain. Sonication raised crossover strain at pH 3, especially at 0.3% (*w*/*w*), but also increased more for pH 5 emulsions, suggesting that ultrasonication not only reinforced the network but also improved its flexibility. These results indicate that 0.3% (*w*/*w*) T-CNC-PE provides an optimal balance between strength and resilience, whereas 0.4% (*w*/*w*) produces more rigid, less flexible gels.

##### Comparison of HB Model Parameters with Viscoelastic Properties

The oscillatory measures (G′, tan δ, crossover strain) and the steady-shear HB parameters (τ_0_, K, n) tell a coherent story about how interfacial organization controls stabilization mechanisms. Lowering the pH to a strong acidic condition (pH 3) reduces electrostatic repulsion between the CNC and improves its interfacial adsorption ability, along with a high detachment energy. Applying ultrasonication results in smaller droplets with greater surface area. Together, these factors increase CNC adsorption and the number of CNC-coated droplet–droplet contacts at pH 3 compared to pH 5. This produces high G′ and low tan δ, and the same conditions generate higher yield stress (τ_0_) and larger K at moderate CNC content (greater resistance after yielding). Notably, for comparison, wood- and cotton-derived CNC or CNF Pickering emulsions with 50–80 wt% oil typically reach G′ on the order of 10^2^–10^3^ Pa only when the particle concentration is ≥1% (*w*/*w*) and, in many cases, after high-pressure homogenization or sonication [11,51,52]. In our T-CNC system, moduli of the same order are obtained at 0.3–0.4% (*w*/*w*) and only with 20 wt% oil under HSE and HSUE, i.e., at least three to fivefold lower nanocellulose loading for a comparable elastic response with lower oil content [21,67,68]. The crossover-strain maximum at the intermediate CNC level mirrors the K optimum at 0.2–0.3% (*w*/*w*) in HSUE. At this concentration, the interface is close to saturation, and the droplet–CNC network is strong yet still capable of internal rearrangement, so it can accommodate deformation without failing early. At the highest CNC loading, excess particles are more likely to form rigid flocs and multi-layer coatings, which restrict structural rearrangement. As a result, the oscillatory response becomes stiffer (G′ increases and tan δ remains low), while K no longer rises and the crossover strain falls slightly, consistent with a firmer but more brittle, less tolerant structure. Storage mainly affects the flow-behaviour index n, especially for HSUE at pH 3, indicating that with time, the network rearranges to shear-thin more readily; this time dependence is subtle in G′ and tan δ.

In mildly acidic conditions (pH 5), the interfacial adsorption ability of the CNC is weaker, along with a lower detachment energy, making the CNC less stable at the interface compared to pH 3. In these conditions, both viscoelastic and HB model parameters agree: G′ is low, tan δ is higher, τ_0_ and K are small, and *n* stays relatively high and unchanged, describing a more fluid-like emulsion that neither resists start-up nor develops strong post-yield structure. These trends differ from those reported for wood- and cotton-derived CNCs, where an elastic-dominant regime (high G′, tan δ < 0.20), appreciable τ_0_, and high K typically occur only at ≥1% (*w*/*w*) and often require sonication [22,53,54]. Storage-induced reductions in *n* are likewise noted mainly at those higher loadings. In our case, similar viscoelastic characteristics are obtained at far lower CNC concentrations, reflecting the stronger interparticle associations and more efficient network formation provided by the high-aspect-ratio T-CNCs [22,53,54,57,68]. The literature showing that ultrasonication reduces droplet size and promotes particle adsorption [51,52,66] supports the elevated G′ and K observed under HSUE, while reports of time-dependent decreases in *n* for plant-CNC emulsions [11,69,70] are consistent with our storage effects. Together, these comparisons indicate that the higher aspect ratio of T-CNCs enables effective interfacial anchoring and bridging at lower particle concentration, allowing the same mechanical regime to be reached at 0.2–0.4% (*w*/*w*) rather than the ≥1% (*w*/*w*) typical of plant-derived CNCs [69,70].

#### 3.2.5. Microstructure and Visual Observation of T-CNC-Stabilized PEs

**Figure 8:**
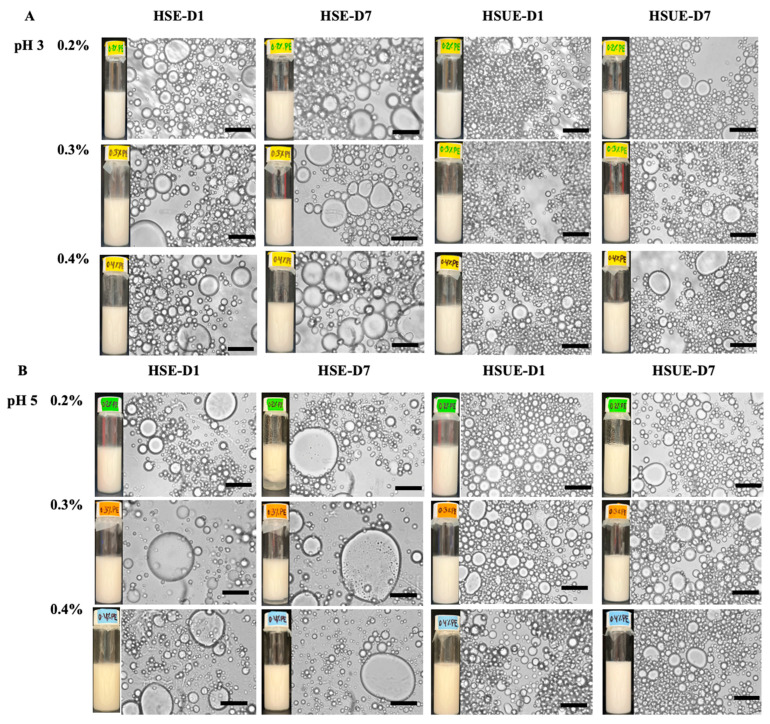
Microstructure and visual observation images of T-CNC-based Pickering emulsions at (**A**) pH 3 and (**B**) pH 5 stabilized with different concentrations of T-CNC (0.2–0.4%, *w*/*w*) on day 1 (D1) and day 7 (D7). Emulsions were prepared by high-shear homogenization (HSE) and by high-shear homogenization followed by ultrasonication (HSUE). Scale bars represent 10 μm.

Visual and microscopic analyses provided complementary evidence of emulsion stability across pH, concentration, and processing conditions (Figure 8). At pH 3, both HSE and HSUE emulsions showed relatively uniform droplets across 0.2–0.4% (*w*/*w*) T-CNC, with no visible phase separation over 7 days. HSUE produced consistently finer and smaller droplets, confirmed by the droplet size distributions and average droplet sizes (Figure 3). Only a minimal droplet growth was observed during storage for HSE, while HSUE remained comparatively stable. These results agree with rheology, where pH 3 emulsions displayed higher viscosity, yield stress, and G′ values, consistent with stronger interfacial adsorption, smaller droplets and reduced coalescence at pH 3. A similar trend was described by Aw et al. [33], who showed that at low CNC concentrations droplet sizes were larger and more polydisperse, while increasing CNC concentration or applying ultrasound yielded finer droplets and improved uniformity, in line with our observations.

At pH 5, visual phase separation was observed in 0.2% and 0.3% (*w*/*w*) T-CNC HSE after 7 days, supported by microscopy showing larger initial droplets and continued growth over time. In contrast, no visual separation was seen for HSUE at the same concentrations, highlighting the stabilizing effect of ultrasonication. However, droplet growth was still evident at 0.2% and 0.4% (*w*/*w*) T-CNC HSUE, indicating very low CNC loading and that after exceeding the surface saturation point they cannot fully suppress coalescence even under improved processing. Similar results were reported by Ma et al. [65,66] who showed that insufficient CNC loading in plant-derived systems led to droplet flocculation and instability, while adequate concentrations produced finer and more stable emulsions. In line with these findings, ultrasonication reduced visible phase separation at low concentrations, but complete suppression of droplet growth required optimal CNC loadings. At 0.4% (*w*/*w*) T-CNC, no phase separation was observed for HSE and HSUE. However, microscopy and droplet size confirmed noticeable droplet growth. This is consistent with Perrin et al. [53,54] who reported that 20 wt% O/W emulsions stabilized by wood CNCs showed droplet growth and phase separation at low CNC concentrations, but required ≥1% (*w*/*w*) CNC to achieve a similar kind of stability, which the T-CNC provided at only 0.2–0.4% (*w*/*w*). Overall, T-CNCs make emulsions more stable at very low concentrations because their long, rod-like shape packs tightly around droplets. Highly acidic pH and ultrasonication strengthen this effect, allowing stable emulsions to form with much less CNC than is usually needed for plant-based CNCs.

## 4. Conclusions

Rod-shaped T-CNC (average length 1694 nm, and width 13 nm, resulting in an average aspect ratio of 122) was extracted using chromium (III) nitrite oxidation from the marine organism tunicate (*Styela clava*). Low concentrations of T-CNC (0.2–0.4%, *w*/*w*) were used to prepare Pickering 20 wt% O/W emulsions at pH 3 and pH 5 using a combination of high-shear homogenization and ultrasonication. At pH 3, the lower-charge T-CNC particles exhibited higher wettability, enabling stronger interfacial adsorption and substantially higher detachment energy than at pH 5 (16,206 kT at pH 3 versus 8808 kT at pH 5). This resulted in emulsions with reduced coalescence, enhanced gel strength, and no visible phase separation over seven days, compared with those prepared at pH 5. Ultrasonication (HSUE) also produced smaller initial droplets than high-shear homogenization (HSE) alone, thereby improving emulsion stability and strengthening the gelled network. Across all conditions, the emulsions behaved as weak gels, with storage modulus exceeding loss modulus. The 0.3% T-CNC pH 3 HSUE formulation exhibited the most desirable rheological profile, including the highest storage modulus, yield stress, consistency coefficient (K), and the lowest flow behaviour index (*n*) and tan δ. Increasing T-CNC concentration to 0.4% did not offer significant improvements (*p* < 0.05). Overall, pH governed particle charge, wettability, and detachment energy, while the emulsification method determined initial droplet size. Together, these factors shaped the rheological and stability characteristics of the emulsions. These findings demonstrate that T-CNC can facilitate the design of Pickering emulsions with tunable rheology and short-term stability at low particle loadings, supporting applications in clean-label foods (such as salad dressings and sauces), cosmetic creams and lotions, and controlled delivery of oil-soluble actives. As a limitation, this study focused on short-term stability (seven days) and on the acidic pH range relevant to targeted applications. Future research should investigate long-term storage behaviour, interactions with real food or cosmetic matrices, and strategies to further refine the T-CNC isolation process to minimize residual chemicals and ensure safe use in consumer products.

## Figures and Tables

**Table 1:** Contact angle, T-CNC particle length and width, interfacial tension, and the calculated values of detachment energy at two pH levels.

pH	Contact Angle (θ)	Length (l), m	Width (q), m	Interfacial $\mathrm{Tension}\;(\gamma_{ow}),$ N/m	Detachment Energy (E_det_), kT or J
pH 3	30°	1694 × 10^−9^	13 × 10^−9^	22.6 × 10^−3^	16,206 kT (6.67 × 10^−17^ J)
pH 5	22°	1694 × 10^−9^	13 × 10^−9^	22.6 × 10^−3^	8808 kT (3.62 × 10^−17^ J)

Note: Interfacial tension at the oil–water interface was measured using the Wilhelmy plate method with a K20 Force Tensiometer (Krüss, Hamburg, Germany), following the procedure reported by Sodhi et al. [50].

## Data Availability

The original contributions presented in the study are included in the article/Supplementary Material, further inquiries can be directed to the corresponding authors.

## Supplementary Materials

The following supporting information can be downloaded at: https://www.mdpi.com/article/10.3390/foods15030509/s1, Figure S1: T-CNC dispersion strain sweeps (top row) and frequency sweep (bottom row) profile at different concentrations (0.2–0.4%) and at pH 3 and pH 5; Table S1: Mean droplet size (D_43_) of the T-CNC-based Pickering emulsions at pH 3 and pH 5 on Day 1 and Day 7 as a function of T-CNC concentration.
