# Supplementary material for: Rheology and Stability of Tunicate Cellulose Nanocrystal-Based Pickering Emulsions: Role of pH, Concentration, and Emulsification Method
Source: Foods. 2026 Feb 1;15(3):509. doi: 10.3390/foods15030509 (PMC12897129; doi:10.3390/foods15030509)
Supplement: Supplementary file 1 [file foods-15-00509-s001.zip › foods-4045421-supplementary.pdf]

Supplementary Data

Rheology and Stability of Tunicate Cellulose Nanocrystal-Based Pickering Emulsions: Role of pH, Concentration, and Emulsification Method

Sumana Majumder, Matthew J. Dunlop, Bishnu Acharya*, Supratim Ghosh*
University of Saskatchewan, Saskatoon, SK S7N 5A9, Canada

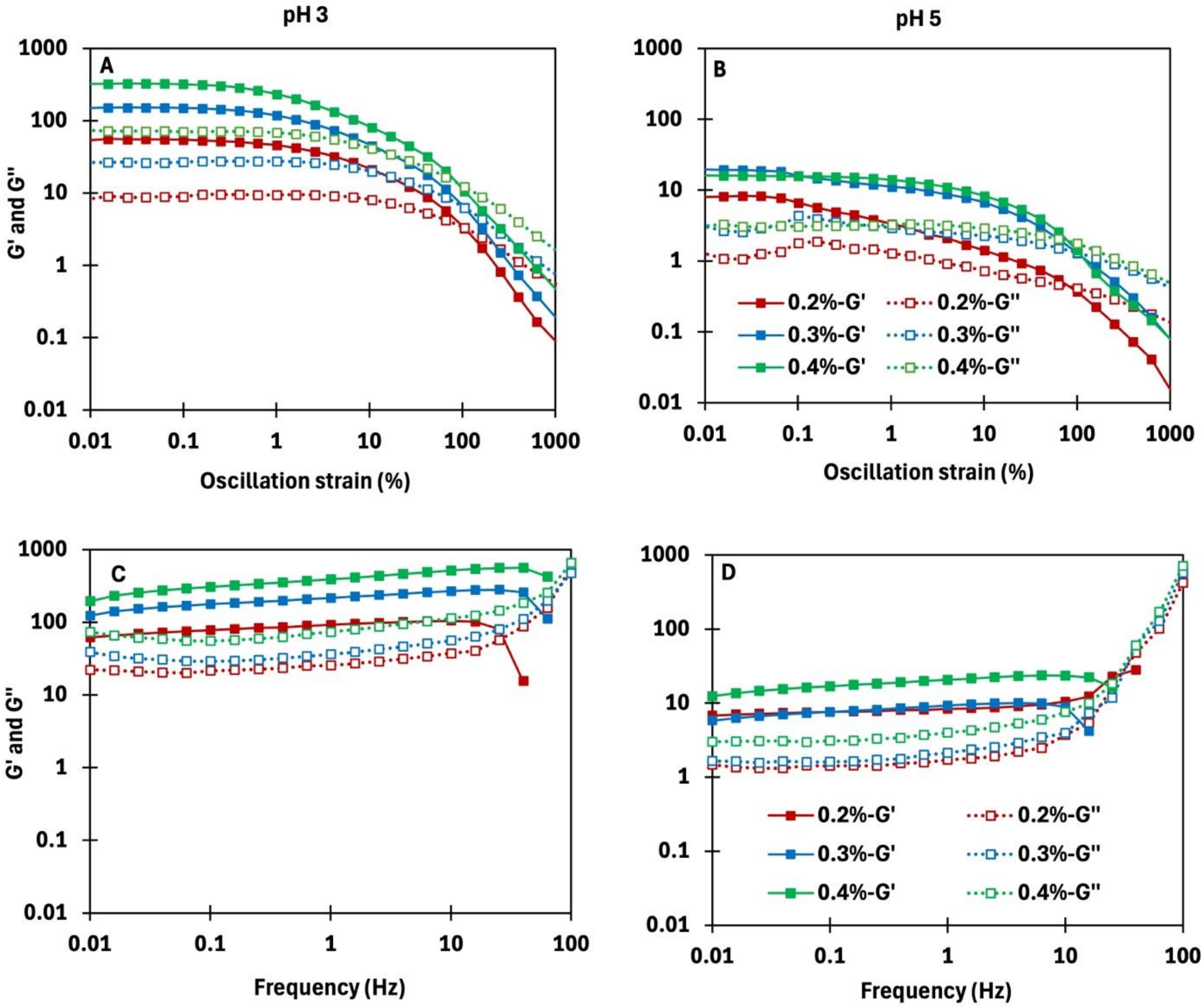

Figure S1: T-CNC dispersion strain sweeps (top row) and frequency sweep (bottom row) profile at different concentrations (0.2-0.4%) and at pH 3 and pH 5.

Supplementary Data

Table S1. Mean droplet size (D_{43}) of the T-CNC-based Pickering emulsions at pH 3 and pH 5 on Day 1 and Day 7 as a function of T-CNC concentration.

Emulsification methods	T-CNC Conc. (%)	pH 3		pH 5	
		Day-1 D_{43} (µm)	Day-7 D_{43} (µm)	Day-1 D_{43} (µm)	Day-7 D_{43} (µm)
**HSE**	0.2	23.1	23.8	57.9	117.7
	0.3	23.6	28.7	75.3	99.1
	0.4	28.4	30.8	90.5	110.0
**HSUE**	0.2	11.6	12.6	25.7	51.2
	0.3	16.2	17.7	8.6	14.0
	0.4	14.0	14.8	9.7	29.1

HSE (High-shear homogenization emulsion), HSUE (High-shear homogenization plus ultrasonicated emulsion)
